# 

**Published:** 1894-06-23

**Authors:** 


**Notices of Births, Deaths, and Marriages** are inserted in this column at a charge of 2s. 6d. for an announcement not exceeding four lines.

**Notice to Advertisers and Subscribers.**

All Advertisements, Orders for Copies of The Hospital, and Business Communications should be addressed to **The Publisher, NOT TO THE EDITOR,** The Hospital, 428, Strand, W.C.

The Cost of Subscription, post free, is as follows:—Per week, 2½d.; per quarter, 2s. 8d.; per half-year, 5s. 3d.; per annum, 10s. 6d. Foreign:—Per Annum, 12s. 8d.; payable, in all cases, in advance.

### NOTICE TO CORRESPONDENTS.

All MS., Letters, Books for Review, and other matters intended for the Editor should be addressed The Editor, The Lodge, Porchester Square, London, W.

The Editor cannot undertake to return rejected MS., even when accompanied by stamped directed envelope.

**"Burdett's Hospital Annual," 1894.**

Fifth year of publication. 900 pp. Boards, gilt lettering. A most complete guide to British, Colonial, Indian, and American Hospitals, Dispensaries, Nursing and Convalescent Institutions, and Asylums. Price 5s. Published by The Scientific Press (Limited), 428, Strand, W.C.

**NOTICE.—The Index for Vol. XV. (ending March 31st, 1894) is on sale at the Office of this Journal, price 2½d., post free.**

### SCRAPS AND GLEANINGS.

A new skin diseases hospital has been added to the City Poorhouse of Aberdeen.

The finances of the Ormskirk Dispensary have been well maintained during the last twelve months.

The Pembrokeshire and Haverfordwest Infirmary has been benefited by a £50 legacy from the late Miss Bullen.

The sanitary arrangements at Christ's Hospital being now complete, the majority of scholars returned to the premises on June 4th.

The Duke and Duchess of Devonshire will visit the Warehousemen, Clerks, and Drapers' Schools, Purley, Surrey, on prize day, June 30th.

Small-pox having broken out at Wednesbury, steps are being immediately taken for the erection of an isolation hospital for the locality.

The Duchess of Teck will present the prizes to the children of the Princess Mary Village Homes at Adlestone on Commemoration Day, July 5th.

An infectious diseases hospital is contemplated for the Ongar Union, Colchester; the local Board of Guardians have already met to discuss plans, &c.

It is announced that the Queen has offered a Knighthood to Dr. J. C. Bucknill, F.R.S., of Bournemouth, in recognition of his services in the Volunteer movement.

A monster bazaar is projected in Dublin for next spring, the funds of which are to be given in favour of the City Orthopædic Hospital, the only one of its kind in Ireland.

The Right Hon. David R Plunket, Q.C., M.P., has been elected Chairman of the Committee of Management of the North-West London Hospital, Kentish Town Road.

H.R.H. the Princess of Wales has become the possessor of one of Messrs. Vigor's mechanical saddles, which we noticed favourably not very long ago in our columns.

A sum of £50 is still due to the treasurer of the Stockton Hospital in order to make up the requisite amount for the scheme of the children's "Free Cots" for that institution.

The annual street collection in aid of the Guest Hospital has just taken place at Dudley. House-to house collections were also made. The total amount realised was £43.

The committee of the Great Northern Central Hospital, Holloway Road, N., have received the sum of £400 from the trustees of Smith's (Kensington Estate) Charity towards the large debt on the maintenance fund.

We learn that the Salters' Company have permanently endowed five beds at Guy's Hospital at a cost of £250 per annum. This bequest is very opportune, as the income of the hospital has fallen considerably lately.

No less than £10,000 was collected at the festival dinner of the Great Ormond Street Hospital for Children. The Duke of York presided, and the Duke of Fife presented an admirable case for this most deserving institution.

The seventh annual parade, promoted by the various friendly and trade societies of Reading, with the beneficent object of helping the Royal Berks Hospital and the convalescent home fund connected with the same, has just been organised.

Nine posts for surgeon-majors are about to be added to Frederick Williams' Institute of Military Medicine. This step will enable doctors to acquire a greater proficiency in certain specialities, as well as in advanced hygiene and bacteriology.

The fever hospital question at Hawick is by no means settled. So much opposition is evinced by the inhabitants, and a largely-attended public meeting has lately been held to protest against the selection of the Netherhall site at Wilton Lodge.

The annual subscription dance in aid of the extension fund of the West London Hospital, took place at the Queen's Hall, Langham Place, W., under the immediate patronage of H.R.H. Princess Louise, Duchess of Fife, and H.R.H. Princess Mary, Duchess of Teck.

The Duke of Norfolk, who is president of the Sheffield Infirmary, has promised to subscribe £1,000 for five years towards the fund being raised to reconstruct the hospital. It is understood that an appeal for funds is shortly to be made to complete the remaining two blocks.

An undertaking in which the Foresters of the Midlands have taken much interest has just reached its climax in the opening of their new convalescent home at Clent. Mr. John Corbett showed his interest in the undertaking by sending a donation of £100 on the occasion.

A long list of subscriptions towards the Cork Eye, Ear, and Throat Hospital testifies to the energy of Lady Arnott and her committee in collecting funds towards the building fund. Sir John Arnott has subscribed £500, and Lady Arnott £100 towards this most deserving scheme.

A meeting of subscribers of the Margate Infirmary for Scrofula was held at Grovesnor House by permission of the Duke of Westminster. The annual report showed that 140 beds out of the 200 had been unoccupied during the year; insufficient pecuniary support having gravely curtailed the good work of the institution.

Miss Winifred Emery (Mrs. Cyril Maude), has sent to the secretary of the Grosvenor Hospital, Vincent Square, Westminster, £82 10s., and the same sum to the Home of the Aged Poor, St. Peter's Square, as the proceeds, after payment of all expenses of the entertainment given by her at the Chelsea Town Hall, on the 21st May.

The Belvedere Hospital for Women at Bath, although only opened in the spring of 1890, and having a capacity alone for six beds, the in-patients have numbered 284, and the out-patients 3,261. The annual expenditure does not exceed much more than £300, a feature in the hospital being that the patients themselves substantially supplement the finances.

We understand that a sum of £1,000 has been promised by the Ecclesiastical Commissioners in London towards the maintenance of the Convalescent Home at Harrowgate. The Commissioners came to this agreement consequent on their being owners of large coal royalties in Durham, from where large numbers of patients have long taken advantage of the home.

### The Student of Medicine.

### APPOINTMENTS.

M. M. Bowlan, M.B.Durh., B.S., D.P.H.Camb., Med. Psych. Cert., appointed Medical Superintendent to St. George-in-the-East Infirmary. E. E. Cass, M.B., B.S.Durh., appointed Medical Officer for the Muncaster District of the Bootle Union. J. Cooper, M.R.C.S.Eng., L.R.C.P.Lond., appointed Casualty Officer and Registrar to the Great Northern Central Hospital. Dr. P. Frankland, F.R.S., appointed Professor of Chemistry in the Mason College, Birmingham. C. W. Glassington, M.R.S.C., L.D.S.Edin., appointed Dental Surgeon to Westminster Hospital. L. M. Guilding, M.A.Oxon., M.B., B.Ch., M.R.C.S.Eng., appointed Medical Officer to the Workhouse of the Reading Union. Dr. W. M. Hamilton, appointed Medical Officer for the Barton District of the Barton-upon-Irwell Union. R. C. Harrison, L.R.C.P.Lond., M.R.C.S.Eng., L.S.A.Lond., appointed Honorary Medical Officer to the Ealing Cottage Hospital. A. W. C. Herbert, L.S.A., appointed Medical Officer for the Sixth District of the Blything Union. Dr. Hood, appointed Medical Officer to the Rattery Parochial Board. J. Norton, M.D., M.R.C.S., appointed Medical Officer of Health to the Vestry of St. Margaret and St. John, Westminster. A. M. Palmer, L.R.C.P.Edin., M.R.C.S.Eng., reappointed Medical Officer of Health to the Whittington Urban Sanitary District. H. L. Palmer, M.R.C.S.Eng., reappointed Medical Officer of Health to the Newtown Urban Sanitary District. E. J. Penny, M.D.Brux., M.R.C.S.Eng., appointed Medical Officer for the First District of the Tonbridge Union. W. S. Pratt, M.D., L.R.C.P. Lond., M.R.C.S., appointed Certifying Factory Surgeon for Rugby and District. W. J. Robertson, M.R.C.S.Eng., L.R.C.P. Lond., appointed House Physician to Charing Cross Hospital. St. John Stanwell, M.B., C.M.Edin., M.R.C.S.Eng., appointed House Surgeon to the Stamford, Rutland, and General Infirmary. J. Steele, L.R.C.P. and S.Edin., reappointed Medical Officer of Health to the Kidsgrove Local Board. T. H. Symons, M.R.C.S. Eng., L.R.C.P.Lond., appointed House Surgeon to Charing Cross Hospital. Dr. W. Thomson, appointed Surgeon and Physician to the British Hospital at Monte Video, South America. G. Templeton, F.R.C.S., L.R.C.P., M.B., C.M.Edin.,

THE STUDENT OF MEDICINE—*continued.*

appointed Junior Medical Officer to the Royal Free Hospital, Gray's Inn Road. E. A. Penam, M.R.C.S., L.R.C.P., M.B. Lond., appointed Junior Resident Medical Officer to the Royal Free Hospital, Gray's Inn Road. Dr. J. W. Dickson, has been elected Anæsthetist to the Grosvenor Hospital for Women and Children, Vincent Square.

### VACANCIES.

The following vacancies are announced this week, the amount of salary in each case being given after the name of the institution:

#### Resident Medical Officers.

Cheltenham General Hospital.—Junior House Surgeon, unmarried, doubly qualified. Salary £40 per annum, with board and apartments. Applications to Mr. F. W. Hayward Butt, Honorary Secretary and Treasurer, by June 23rd.

Darenth Schools for Imbeciles, near Dartford, Kent.—First Assistant Medical Officer, doubly qualified. Salary £160 per annum, rising £20 annually to £200, with board, furnished apartments, attendance, and washing. Applications, on forms to be obtained at the office of the Board, Norfolk House, Norfolk Street, Strand, W.C., to T. Duncombe Mann, Clerk to the Board, at the offices, by June 23rd.

General Infirmary, Gloucester, and Gloucestershire Eye Infirmary.—Assistant House Surgeon. Appointment for six months, eligible for re-election. No salary, but board, residence, and washing provided. Applications to H. P. Pike, Secretary, by June 27th.

Newport and Monmouthshire Infirmary, Newport, Mon.—House Surgeon, doubly qualified. Salary £100 per annum, with board and residence. Applications to the Secretary by June 23rd.

Royal Albert Edward Infirmary, Wigan.—Junior House Surgeon. Salary £80 per annum, with apartments, rations, and washing. Applications and testimonials to Will. Taberner, General Superintendent and Secretary, before July 25th.

Sunderland Borough Lunatic Asylum.—Medical Superintendent, doubly qualified. Salary £350 per annum, with furnished house, board for self and wife (if married), washing, coals, light, two servants, and use of garden. Applications, endorsed "Medical Superintendent," to Fras. M. Bowey, Clerk to the Visiting Committee, Town Hall, Sunderland, by June 30th.

West Riding Asylum, Wakefield.—Two Resident Clinical Assistants. Appointment for six months. No salary, but board, residence, and attendance. Applications and testimonials to the Medical Director.

Wolverhampton and Staffordshire General Hospital, Wolverhampton.—Resident Assistant. Appointment for six months. Applications, inscribed "Application for Resident Assistant," to the Chairman of the Medical Committee, by June 25th.

#### Non-Resident Medical Officers.

Boyle Union, Ballinameen Dispensary.—Medical Officer. Salary £120 per annum, with £10 yearly as Medical Officer of Health, together with vaccination and registration fees. Applications to Mr. T. A. Cox, Honorary Secretary, Hermitage, Croghan. Election on June 25th.

Kama Hospital, Bombay.—Lady Doctor as First Physician. Salary Rs. 700, rising by annual increments of Rs. 40 to Rs. 900 per annum. First-class passage to Bombay provided. Applications to the Secretary, Public Department, India Office, London, S.W., by June 20th.

Royal London Ophthalmic Hospital, Moorfields.—Curator, non-resident. Appointment for one year; renewable. Salary £120 per annum. Applications to the Secretary by June 30th.

Salters' Company.—Research Fellowship in Experimental Pharmacology. Annual value of £100, and is tenable in the Medical School of St. Thomas's Hospital. Applications to the Secretary to the Medical School, St. Thomas's Hospital, S.E., before June 30th.

Salters' Company.—Research Fellowship in Chemistry. Annual value of £100, tenable in the Research Laboratory of the Pharmaceutical Society. Applications to Professor Dunstan, F.R.S., Director of the Research Laboratory of the Pharmaceutical Society, 17, Bloomsbury Square, W.C., before June 30th.

University of Edinburgh.—Chemical Assistant to Professor of Physiology. Salary £180 per annum. Applications to the Secretary of the University Court before July 1st.

### The Hospital Advertiser.

Norfolk House, Norfolk Street, Strand, W.C.,

### THE HOSPITALS ASSOCIATION,

### 428, STRAND, LONDON, W.C.

The following Pamphlets constitute a valuable collection, full of interest to all workers among the Sick Poor.

### SPECIAL HOSPITALS.

**Foundling Hospitals at Home and Abroad.** By Mr. S. Osborn, F.R.C.S., Soho Hospital for Women.

**The Origin. History, Work, and Present State** of Metropolitan Lying-in Hospitals. By Mr. Thomas Ryan, Secretary to St. Mary's Hospital, Paddington.

**Hospitals for the Insane and Clinical Instruction** in Asylums. By Mr. Richard Green, F.R.C.P.

**Epileptic Colonies.** By Mr. C. Theodore Ewart.

### HYGIENE AND DIET.

**Disinfection, Hospital and Domestic.** By Mr. A. Wynter Blyth, Medical Officer of Health for St. Marylebone.

**Cholera and the Hospitals.** By Dr. J. C. Steele.

**Leprosy and Leper Houses.** By Sir William Moore, K.C.I.E.

**Thoughts on the Diet of Nurses in Hospitals** and Infirmaries. By Miss L. Twining.

**The Use of Alcohol in Hospitals.** By Mr. George Sturge.

**Nurses' Food, Work, and Recreation.** By Mr. Henry C. Burdett, Deputy Chairman of "The Hospitals Association," &c.

### HOSPITAL FINANCE.

**The Hospital Sunday and Saturday Funds.** By Mr. Henry C. Burdett.

**Should Public Charities Pay Local Rates?** By Mr. J. S. Wood, late Secretary, Chelsea Hospital for Women.

**Should Charities Pay Rates?** By Mr. G. A. Cross, Secretary, London Homœopathic Hospital.

**Sixteen Years of Hospital Sunday,** its Influence upon the Metropolitan Medical Charities. By Sir Sydney H. Waterlow, Bart.

**Hospital Extravagance and Expenditure.** By Mr. P. Michelli, Secretary to the Seamen's Hospital, Greenwich.

**Contributions by Patients in Relation to the** Financial Condition of London Hospitals. By Mr. W. Burdett-Coutts, M.P.

**Some Proposals for Contributions by Patients** to Hospitals. By Mr. H. Nelson Hardy, F.R.C.S.

**The Chronic Indigence of our Hospitals.** By Mr. B. Burford Rawlings, Secretary to the National Hospital, Queen Square, W.C.

### AMBULANCE AND THE TRANSPORT OF SICK AND INJURED PERSONS.

**Hospital Ships.** By Dr. P. Murray Braidwood.

**Horse Ambulances in Connection with Hospitals.** By Captain William Joynson, Chairman of the Northern Hospital, Liverpool.

**The Ambulance and Hospital Arrangements** of an English Army in the Field. By Surgeon-Major Evatt, R.M.A., Woolwich.

**The Conveyance of Injured Persons to the** Metropolitan Hospitals. Defects in the existing system and proposals for their removal. By Mr. Thomas Ryan, Secretary to St. Mary's Hospital, W.

### NURSING.

**How far should our Hospitals be Training** Schools for Nurses? By Dr. J. S. Bristowe, F.R.S., and Miss C. E. Lückes, Matron of the London Hospital.

**Queen Victoria's Jubilee Hospital.** The Object and the Work. By the Rev. the Master of St. Katharine's.

**Is the Nursing at the London Hospitals Sectarian?** By Dr. G. W. Potter.

**Nursing in Workhouse Infirmaries.** By Mr. S. Benton, M.R.C.S., Central London Sick Asylum and Highgate Infirmary.

**The Registration of Nurses.** By Dr. J. C. Steele.

**Report of the Joint-Sectional Committee on** Registration.

**Cottage Nurses and their Training.** By Miss Broadwood.

**Nursing in Workhouses, and Workhouse Infirmaries.** By Miss Wilson, Hon. Sec., "Workhouse Infirmary Nursing Association."

**The Registration of Midwives.** By Mrs. Henry Smith (President of the Midwives' Institute) and Dr. Leith Napier, M.R.C.P,

### HOSPITAL ARCHITECTURE.

**Hospitals in Relation to the Public Health.** By Professor John Marshall, F.R.S.

**The Miller Memorial Hospital at Greenwich.** By Mr. Keith D. Young, F.R.I.B.A.

**Hospital Construction.** By Mr. Keith D. Young, F.R.I.B.A.

**The Ventilation of Hospitals.** By Dr. C. T. Williams, M.A., Brompton Consumption Hospital.

**A Model Hospital.** The New General Hospital, Hamburg. By Mr. C. W. Thies, Sec. Royal Free Ho

**An Inquiry into the Structural Arrangements** of the Nursing Departments of Hospitals. By Mr. H. Saxon Snell, and

**Hospital Ventilation, with Special Reference to** the Victoria Infirmary, Glasgow. By Dr. J. C. Steele.

### ADMINISTRATION.

**The Hospital Problem—Hospital Income, Expenditure,** Bookkeeper's Audit. By Mr. Henry C. Burdett.

**The Legal Restrictions on Gifts to Charity.** By Mr. L. S. Bristowe, M.A., Barrister-at-Law.

**The Mortmain and Charitable Aids Act, 1891.** By Mr. L. S. Bristowe, M.A., Barrister-at-Law.

### HOSPITAL OUT-PATIENT QUESTION.

**Difficulties Associated with the Administration** of the Out-Patient Department, and How Best to Deal with Them. By Mr. W. J. Nixon, House Governor of the London Hospital.

**Is it Desirable that Hospitals Should be Made** Self-supporting, and if so, to what Extent? By Rev. Canon E. Clarke, Bolingbroke House Pay Hospital.

**What is the Best Plan for Establishing Provident** Dispensaries in Due Relation to the Hospitals? By Mr. C. Radley, late Sec. of the Met. Provident Med. Assoc.

**A Re-discussion of the Out-Patients Question.** By Dr. J. S. Bristowe, F.R.S.

**Report of a Conference with a Deputation** from the Met. Branch of the Brit. Med. Assoc.

**The Best Means of Preventing the Abuse of** Hospital Charity. By Dr. W. Collier, M.A., Radcliffe Infirmary, Oxford.

**Some Proposals for Contributions by Patients** to Hospitals. By Mr. H. Nelson Hardy, F.R.C.S.

### GENERAL.

**The London Poor and Their Medical Needs.** By Mr. Conrad W. Thies, Secretary Royal Free Hospital.

*Copies of the above may be had on application to the Hon Sec. as above; price 6d. each.*

